# Facial Affective Behavior in Borderline Personality Disorder Indicating Two Different Clusters and Their Influence on Inpatient Treatment Outcome: A Preliminary Study

**DOI:** 10.3389/fpsyg.2020.01658

**Published:** 2020-07-29

**Authors:** Gerhard Dammann, Myriam Rudaz, Cord Benecke, Anke Riemenschneider, Marc Walter, Monique C. Pfaltz, Joachim Küchenhoff, John F. Clarkin, Daniela J. Gremaud-Heitz

**Affiliations:** ^1^Psychiatric Hospital, University of Basel, Basel, Switzerland; ^2^Münsterlingen Psychiatric Hospital, Münsterlingen, Switzerland; ^3^University Hospital for Psychiatry and Psychotherapy, Paracelsus Medical University, Salzburg, Austria; ^4^Department of Family and Child Sciences, Florida State University, Tallahassee, FL, United States; ^5^Institute of Psychology, Clinical Psychology, University of Kassel, Kassel, Germany; ^6^Department of Psychiatry, University Hospital Zurich, Zurich, Switzerland; ^7^Personality Disorders Institute, Cornell University Medical School, New York, NY, United States

**Keywords:** borderline personality disorder, facial affective behavior, treatment outcome, EMFACS, psychopathology, personality organization

## Abstract

**Background:**

The purpose of the present study was threefold: first, to investigate the facial affective behavior in patients with a borderline personality disorder (BPD); second, to examine whether these patients could be divided into clusters according to facial affective behavior; and third, to test whether these clusters would influence the inpatient treatment outcome.

**Methods:**

Thirty inpatients with BPD were assessed with the Structured Clinical Interviews for DSM-IV Axis I and II Disorders (SCID I, SCID II) and had to complete a series of questionnaires before and directly after the 12-week long inpatient treatment. Facial affective behavior was recorded during the structured interview for personality organization (STIPO) and afterward coded with the emotional facial action coding system (EMFACS). Measures on psychopathology [beck depression inventory (BDI), Spielberger state and trait anxiety inventory (STAI), Spielberger state and trait anger inventory (STAXI), and symptom cheklist-90-revised (SCL-90-R)], interpersonal problems [Inventory of Interpersonal Problems (IIP)], and personality organization [inventory of personality organization (IPO)] were administered.

**Results:**

Cluster analysis before the treatment yielded two groups that differed in general facial expressivity, and regarding the display of anger, contempt, and disgust. The effect sizes of the repeated measures ANOVAs showed that persons with higher scores on the affective facial expressions benefitted more from the treatment in terms of STAI state anxiety, STAXI state and trait anger, IIP total, and the two scales primitive defenses and identity diffusion of the IPO, whereas persons with lower scores benefitted more on the scale IPO reality testing.

**Conclusion:**

Our results indicated some initial trends for the importance of facial affective behavior in patients with BPD and their treatment outcome.

## Introduction

Borderline personality disorder (BPD) is one of the most common personality disorders, affecting about 0.5–5.9% of the general population, around 10% of psychiatric outpatients, and 15–20% of psychiatric inpatients (Lenzenweger et al., 2007; Trull et al., 2010). Its main characteristics include affective instability, disturbed identity, impulsivity, and interpersonal problems (Lazarus et al., 2014). Inappropriate anger or difficulties controlling anger can also be present in BPD (Lis and Bohus, 2013). According to the model of personality organization (Kernberg, 1984), impaired primitive defenses centering around splitting and identity diffusion dominate in borderline personality organization, whereas reality testing is preserved. BPD patients also show high rates of comorbidity with depressive and anxiety disorders (e.g., Sauer-Zavala et al., 2016).

Facial affective display or behavior can be seen as a relationship regulation tool: it serves to create specific relationship patterns by communicating distinct affective information. Thus, non-verbal affective processes play an important role in maintaining mental disorders (Benecke et al., 2005). There are several studies comparing differences in facial affective behavior between clinical and non-clinical populations (Davies et al., 2016). Researchers found reduced facial affective behavior for several disorders including schizophrenia, obsessive-compulsive disorder, bipolar disorder, posttraumatic stress disorder (PTSD), and major depression (MD; Gehricke and Shapiro, 2000; Renneberg et al., 2005; Krause and Kirsch, 2006; Bersani et al., 2012, 2013), as well as more negative affects for PTSD (Kirsch and Brunnhuber, 2007). In contrast, Peham et al. (2015) did not find significant differences in facial affective behavior between diagnostic groups.

Research comparing patients with BPD is only sparse: Krause (1998) found in single case studies predominantly disgust in the facial expressions of his BPD patients. Facial expressions of disgust are also very pronounced in phobic patients when they are confronted with the phobic object (De Jong et al., 2002; Cisler et al., 2009). Peham et al. (2015) understand facial disgust as a general expression of aversion. Renneberg et al. (2005) compared female patients with BPD, MD, and a non-patient female control group in their facial affective response to positive and negative mood-inducing movie clips. BPD patients and patients with MD had lower overall facial expressiveness than healthy controls. The clinical groups did not differ in their facial responses to negative mood induction. Depressed patients showed fewer Duchenne smiles (happiness) in response to positive film clips than BPD patients did. Duchenne smile is usually seen as an expression of feelings of happiness, whereas social smile is seen as a voluntary social signal without necessarily being associated with an inner experience of a positive feeling. Additionally, BPD patients showed significantly less surprise expressions than the control group.

Buchheim et al. (2007) compared the facial affective behavior of patients with BPD and a non-patient control group during the adult attachment projective interview (AAP; George and West, 2003). BPD patients showed more facial disgust and social smiling but less contempt than the non-patient group. Attachment style influenced these results: facial behavior related to disgust was found to be prominent in BPD patients with unresolved trauma. Likewise, contempt was mainly shown by persons with unresolved trauma in the non-patient control group.

In a naturalistic pilot study, Benecke and Dammann (2004) analyzed the facial affective behavior of 13 BPD patients. As expected, BPD patients very often showed anger, contempt, and disgust (with partially extremely high values especially for contempt), but they also often showed happiness in a clinical interview. Fear, surprise, and sadness were rare. They found that the facial affective behavior of their patients showed a high variance, and thus, they conducted a cluster analysis. A hierarchical cluster analysis revealed two clusters of facial behavior in the borderline sample: in the first cluster, facial affective expressions of anger, contempt, and disgust dominated, with disgust being the most frequently shown affect (lead affect). The second cluster showed less overall facial affective activity and less surprise, fear, anger, and disgust compared to Cluster 1. The lead affect in the second cluster was Duchenne smile.

Daros et al. (2016) showed that participants were able to distinguish women with a BPD from a matched non-psychiatric control group based on facial cues from photos. However, Peham et al. (2015) failed to find significant differences in the frequency of facial affective behavior of BPD patients compared to patients with other disorders and healthy controls. Bock et al. (2016) distinguished different functions of facial affective expressions, depending on the “target” of the affect. They found that different patient groups did not differ in the total amount of negative affective facial display (same sample as in Peham et al., 2015), but that patients with low level of structural integration, like BPD patients, directed their aggressive affects more often toward the interviewer (“interactive function”) and toward the whole self as well as to whole object representations (in contrast to single aspects of the self or single aspects of objects). The results of Gebhardt et al. (2016) point into a similar direction: patients who see themselves less disturbed than external raters are more likely to display a high amount of disgust combined with gazing contact toward the interviewer, indicating that these patients induce negative relationship patterns. Moreover, Brüne et al. (2015) compared the non-verbal behavior as measured with the Ethological Coding System for Interviews in BPD patients and controls during two clinical interviews, one under oxytocin and one under placebo. They found that patients with BPD showed less affiliative behavior (i.e., behaviors that invite and positively reassure social interactions such as “head to side” movements) in the OT condition compared to controls, even though they showed less flight behavior (i.e., behaviors that lead to cutting of communication such as “look away”) in the oxytocin condition compared to placebo.

Hepp et al. (2018) showed that individuals with BPD were rated more negatively than healthy controls based on video sequences in which the participants spoke about their personal preferences (e.g., books, food, and hobbies) and their behavior in an economic game (i.e., participants were asked to divide a certain amount of money between themselves and an unknown person). The two rater samples were exposed to these videos for the first time without knowing anything about the participants (i.e., zero-acquaintance). Specifically, the individuals with BPD were seen in the video sequences as less trustworthy and likeable than the healthy controls in both rater groups. In addition, in one rater group, the individuals with BPD were rated as less cooperative in the economic game, although both groups shared the same amount of money. Moreover, the effects were larger when the videos were presented without the audio trace suggesting that the raters must have relied on visual cues rather than the speech content and prosody to form their judgments. The authors conclude that low expectations of likeability, trustworthiness, and cooperation in persons with BPD could lead to problems in social interactions in the future and therefore maintain the disorder by strengthening their dysfunctional beliefs that they will be rejected and have to protect themselves to prevent negative events. In a subsequent study, Hepp et al. (2019) examined which cues (i.e., positive affect display, negative affect display, and eye contact) raters could have used to form their negative judgments about individuals with BPD. Individuals with BPD were rated as showing less positive affect and more negative affect than the healthy controls. In addition, low positive affect display mediated the association between BPD and negative evaluations (i.e., low likeability and trustworthiness).

In sum, the result indicated that patients with BPD or patients with a low level of structural integration show more negative facial affective behavior (in particular, disgust) or less positive affect as well as less affiliative behavior than healthy controls (e.g., Buchheim et al., 2007; Brüne et al., 2015; Bock et al., 2016; Hepp et al., 2019). Moreover, the patients with BPD were rated more negatively than healthy controls based on visual cues during first acquaintances (Hepp et al., 2018). Based on these findings and findings from other studies on the association between facial appearance and suicidality (Kleiman and Rule, 2012), it could be argued that facial affective displays may be useful in the clinical context. For instance, a lack of facial displays could signal psychopathology and could contribute to shortcomings in social interactions and thus maintain the disorder. While there is evidence that facial affective behavior can predict the severity of depressive disorders over the course of treatment (e.g., Harati et al., 2019), we are not aware of any study that examined the influence of facial affective behavior on treatment success in BPD. The purpose of this study was therefore to close this gap. Because of the previously reported broad repertoire of invariant emotional facial expressions in BPD, we first investigated whether the results of the pilot study of Benecke and Dammann (2004) regarding two different facial affective clusters could be replicated. In a next step, we tested whether the clusters influenced the inpatient treatment outcome (i.e., personality organization, interpersonal problems, and psychopathology). We did not formulate specific hypotheses due to the exploratory nature of the study.

## Materials and Methods

### Study Design and Participants

A total of 55 patients were asked to participate in the study. Of these, 37 agreed to participate in the study. All patients were inpatients at the Psychiatric Hospital of the University of Basel and were diagnosed with BPD according to the DSM-IV-TR criteria. Patients participated in an inpatient study for BPD patients [Basel Borderline Inpatient Study (BABIS)] and were treated at a specialized psychotherapeutic unit with a set stay length of 12 weeks, based on empirically validated treatment manuals “transference-based psychotherapy (TFP)” and “dialectical behavior therapy (DBT).” The aims of the BABIS were to compare the effects of this specialized treatment versus treatment as usual (i.e., non-specific psychotherapy, psychoeducation in group therapy, supportive talks with staff nurses, and individual sessions with a social worker) and to identify the possible influence of subgroups within the heterogeneous group of BPD patients. The specialized inpatient treatment combined individual TFP sessions (in accordance with the TFP treatment manual; see Clarkin et al., 2006) with TFP-oriented psychodynamic group therapy with nurses and a social worker. Whereas DBT skills sessions focus particularly on mindfulness and on coping with extreme affect states and dysfunctional behavior, TFP targets the conflicts among the patient’s internal representations of self and others within the transference and interpersonal problems (Yeomans et al., 2012). Depending on the demonstrated improvements in affect regulation (Linehan et al., 2006), patients attended additionally DBT-based skills-training groups conducted by trained staff nurses. Psychopharmacologically experienced psychiatrists prescribed the medication. All patients were on medication deemed appropriate by the psychiatrists and in accordance with the recommended APA guidelines (Soloff, 2000). Detailed descriptions of the aims, methods, disorder-specific-based settings, and sample characteristics and other results of the BABIS have been reported separately (Sollberger et al., 2012; Agarwalla et al., 2013; Gremaud-Heitz et al., 2014; Dammann et al., 2016). Exclusion criteria were being diagnosed with schizophrenia, schizoaffective disorder, active psychosis, or acute manic episode. Assessments were conducted during the first week after entering the clinic (pre-assessment) and 12 weeks after the initial assessment (post-assessment).

### Ethical Standards

Written informed consent was obtained from each patient. The study was approved by the local Ethics Committee (EKBB). Participants were not compensated for participation.

### Interviews

Clinically experienced interviewers received instruction on the Structured Clinical Interviews for DSM-IV Axis I Disorders (SCID-I/P; First et al., 1996) and for DSM-IV Axis II Disorders (SCID-II; First et al., 1997) and were trained to pay particular attention to distinguishing Axis I mental state conditions from Axis II personality trait phenomena. The SCID I and II are semi-structured interviews for assessing clinical and personality disorders. Good interrater reliability (SCID I mean Kappa 0.71; SCID II mean Kappa 0.84) has been shown for both interviews (Lobbestael et al., 2011).

### Questionnaire Data

To assess borderline features, the inventory of personality organization (IPO; Kernberg and Clarkin, 1995; Dammann et al., 2002) was used. In the present study, the three primary scales of the IPO were used, namely, identity diffusion, primitive defenses, and reality testing. Good validity and reliability have consistently been demonstrated for the IPO (Lenzenweger et al., 2001). For evaluation of interpersonal criteria, we used the German version of the inventory of interpersonal problems (IIP; Horowitz et al., 2000), a 64-item self-report instrument designed to measure interpersonal deficiencies. The validity and reliability of the IIP have been demonstrated (Horowitz et al., 2000). In the current study, the global score was used. To measure the general psychiatric symptoms and subjective complaints, we administered the German versions of the symptom checklist-90-revised (SCL-90-R; Franke, 1995), the beck depression inventory (BDI; Hautzinger et al., 2000), the Spielberger state and trait anxiety inventory (STAI; Laux et al., 1981), and the Spielberger state and trait anger inventory (STAXI; Schwenkmezger et al., 1992).

### Coding of Facial Affective Behavior

Facial affective behavior was coded with the emotional facial action coding system (EMFACS; Ekman and Friesen, 1978; Ekman et al., 1978), a short version of the Facial Action Coding System (FACS; Ekman et al., 2002a, b), which concentrates on emotion-relevant facial movements. The FACS is a widely used standardized instrument for the analysis of facial expressions based on the anatomy of facial movements. Each distinguishable visible action of facial muscles is assigned to a single action unit (AU). With a so-called “lexicon,” facial expression codings are assigned to affect categories: *anger, disgust, contempt, fear, sadness, surprise*, and *smile*. In addition, there is a set of rules that permit distinguishing between “felt” joy expressions (*Duchenne smile*; AUs 6 + 12) and “social” smiles (AU 12 only). In recent studies concerning interrater reliability of FACS codings, good to very good values were found (Sayette et al., 2001; Peham et al., 2015). All raters of the Facial Action Coding System were trained and showed good interrater reliability: Kappa = 0.931 for the identification of facial events and Kappa 0.898 for the coding of basic emotions. This is similar to the interrater reliability in other studies on facial affective behavior (e.g., Merten, 2005).

Facial affective behavior of the patients was recorded during a face-to-face interview on personality organization, the structured interview for personality organization (STIPO; Clarkin et al., 2004; Doering et al., 2013). The STIPO is a semi-structured instrument to assess personality organization basing on Kernberg’s psychodynamic concept (Kernberg, 1984) postulating three levels of personality organization: neurotic, borderline, and psychotic. On a dimensional level identity, object relations, primitive defenses, coping/rigidity, aggression, moral values, and reality testing are determined. The facial affective behavior was coded from tape. The two interviewers of the STIPO interview were trained and showed good interrater reliability. The interviews were coded by psychologists certified for the EMFACS (not by the interviewers).

### Statistical Analyses

All statistical analyses were conducted with SPSS/22.0. Based on the utilized EMFACS categories at pre (see Table 1), a hierarchical cluster analysis was conducted. We used the average linkage method and squared Euclidean distance measure. Values were transformed to *z*-scores prior to analysis. One person was excluded from the cluster analysis due to extremely high general facial activity at pre (more than 2.5 standard deviations above the mean). The intraclass correlations were calculated using the package nlme in R. Correlation analyses were run for the two clusters separately. However, due to the small sample size of cluster 2, only the effect sizes and not the *p*-values were interpreted (e.g., Sullivan and Feinn, 2012). Independent samples *t*-tests were done to compare differences between the general facial activity and the primary affects between the clusters at pre. Furthermore, we run several repeated measures ANOVAs to test whether the clusters differed on the treatment outcome, namely psychopathology, interpersonal problems and personality organization. Because small sample sizes increase the likelihood of a Type II Error, the effect sizes were interpreted in the present study. The effect sizes were classified as follows: *r* = 0.1 indicated a small effect, *r* = 0.3 indicated a medium effect and *r* = 0.5 indicated a large effect, $\eta_{\text{p}}^{2}$ = 0.01 indicated a small effect, $\eta_{\text{p}}^{2}$ = 0.06 indicated a medium effect, and $\eta_{\text{p}}^{2}$ = 0.14 indicated a large effect.

**TABLE 1:** Used categories of the EMFACS.

Categories	Explanations
(1) Activity	Facial activity
(2) Primary affects	Summary category of all primary affects (3–9)
(3) Anger	
(4) Contempt	
(5) Disgust	
(6) Fear	
(7) Sadness	
(8) Surprise	
(9) Duchenne smile	Felt smile
(10) Social smile	
(11) Possible smile	
(12) Anger/disgust	
(13) Fear/surprise	
(14) Happy/fear	
(15) Happy/contempt	
(16) Happy/anger	
(17) Happy/disgust	
(18) Happy/surprise	
(19) Possible anger	
(20) Unspecific negative	
(21) Possible fear	
(22) AU 20	Fear fragment
(23) Illustrators	(AU 1 + 2)

*EMFACS, emotional facial action coding system; AU, action unit.*

## Results

### Preliminary Analyses

Thirty-seven patients diagnosed with BPD were included in the study and interviewed. Five patients did not complete the questionnaires and were therefore excluded. Two patients did not finish the second part of our study and were also excluded. Of the 30 patients included in the study, 28 (93.3%) were female and 2 (6.7%) male. The mean age was 30.8 years (SD = 6.1; see Table 2). Twenty-eight patients (93.3%) were diagnosed with a comorbid Axis I Disorder, most frequently with an affective disorder (*n* = 26, 86.7%); anxiety disorders were diagnosed in 18 patients (60%). Eighteen patients (60%) showed a comorbid Axis II disorder, predominantly a Cluster C disorder (*n* = 16, 53.3%). With regard to the facial affective behavior, the patients expressed high amounts of disgust and contempt but also social smiles. The results are presented in Table 2.

**TABLE 2:** Demographic and clinical characteristics of patients with BPD (*n* = 30).

Age, mean (SD)	30.1 (9.5)
Gender, *n* (%)	
Male	2 (6.7)
Female	28 (93.3)
Marital status, *n* (%)	
Living alone	24 (80)
Living with a partner	6 (20)
Current employment, *n* (%)	
Employed (full/part time)/apprenticeship	17 (56.7)
Unemployed/disability pension	13 (43.3)
Years of education, *n* (%)	
<9 years	12 (40)
9–12 years	11 (36.7)
>12 years	7 (23.3)
Duration of BPD, *n* (%)	
<1 year	2 (6.7)
<1–5 years	9 (30)
5–10 years	5 (16.7)
10–20 years	9 (30)
>20 years	5 (16.7)
Comorbid Axis I Disorder *n* (%)	
None	2 (6.7)
Affective disorder	26 (86.7)
Anxiety disorder	18 (60)
Substance-related disorder	17 (56.7)
Eating disorder	12 (40)
Comorbid Axis II Disorder *n* (%)	
None	12 (40)
Cluster A	3 (10)
Cluster B	3 (10)
Cluster C	16 (53.3)
Facial affective behavior at pre, mean (SD)	
Anger	7.41 (8.06)
Contempt	14.84 (19.10)
Disgust	79.71 (69.06)
Fear	1.32 (3.25)
Sadness	4.98 (7.77)
Surprise	0.88 (2.10)
Duchenne smile (true smile)	8.78 (17.57)
Social smile	42.03 (46.42)

*BPD, borderline personality disorder; SD, standard deviation.*

### Main Analyses

#### Cluster Analysis and Facial Affective Behavior

The cluster analysis resulted in two clusters in which patients could be divided into. As Table 3 shows, patients of Cluster 1 exhibited higher values in the general facial activity as well as in each primary affect. However, there were only significant differences for the general facial activity and the negative affects anger, contempt, and disgust. Mean values of the Cluster 1 group for anger, contempt, and disgust were more than three times higher than in the Cluster 2 group. Also, both clusters showed relatively high amounts of social smile.

**TABLE 3:** Means and standard deviations of the facial affective behavior for the two clusters at pre.

Variable	Cluster 1 (*n* = 20)		Cluster 2 (*n* = 9)		Cluster 1 vs. Cluster 2		ICC
			
	*M*	SD	*M*	SD	*T*	*p*	
General activity	569.04	128.44	255.00	79.39	6.740	0.000	0.78
Anger	9.54	8.84	2.16	2.21	3.496	0.002	0.29
Contempt	16.55	16.80	4.04	4.14	3.126	0.005	0.29
Disgust	92.16	60.37	30.75	31.03	3.611	0.001	0.37
Fear	1.57	3.73	0.17	0.51	1.644	0.115	0.02
Sadness	6.11	9.01	2.18	3.63	1.673	0.106	0.04
Surprise	1.23	2.48	0.00	0.00	1.467	0.154	0.08
Duchenne smile	9.09	20.58	7.39	10.28	0.234	0.817	0.00
Social smile	48.47	50.65	21.96	28.71	1.459	0.156	0.08

*M, mean; SD, standard deviation; ICC, intraclass correlation.*

#### Correlations Between Facial Affective Behavior and the Study Variables

The correlations between the facial affective behavior and the study variables at pre are presented in Table 4 separately for the two clusters. In Cluster 1, the highest correlations resulted for general facial activity and STAXI state anger plus social smile and STAXI trait anger, respectively. Specifically, a medium positive correlation resulted between general facial activity and STAXI state anger, meaning that patients who showed high general facial activity also had high levels of state anger. Furthermore, there was a medium negative correlation between social smile and STAXI trait anger indicating that people who showed less social smiles also had high levels of trait anger. In Cluster 2, the highest correlation resulted for general facial activity and the STAI state anxiety. Specifically, there was a large negative correlation between the general facial activity and the STAI state anxiety. This implies that people who showed low general facial activity had high levels of state anxiety. Also a large negative correlation was found between the general facial activity and the BDI. Furthermore, there were large negative correlations between some primary affects and the study variables: Anger and STAI state anxiety, contempt and STAI state anxiety, fear and BDI, fear and IPO primitive defenses as well as IPO identity diffusion, and sadness and BDI. Finally, there was a large positive correlation between disgust and STAXI trait anger.

**TABLE 4:** Correlations between the facial affective behavior and the study variables for the two clusters at pre.

Variable	Cluster 1 (*n* = 20)								
	General activity	Anger	Contempt	Disgust	Fear	Sadness	Surprise	Duchenne smile	Social smile
SCL GSI	0.40	–0.28	–0.01	0.05	0.26	0.17	0.28	–0.21	–0.13
STAI state anxiety	0.12	–0.14	0.08	0.07	0.13	0.24	0.18	–0.31	–0.02
STAI trait anxiety	0.29	0.02	–0.17	0.10	–0.07	–0.16	0.02	–0.31	0.20
STAXI state anger	0.45*	0.09	0.05	0.19	0.30	0.08	0.18	–0.07	–0.20
STAXI trait anger	0.31	0.06	0.14	0.24	0.27	0.39	0.34	–0.26	−0.48*
BDI	0.26	–0.08	0.00	0.25	–0.25	–0.25	–0.31	–0.30	0.05
IIP total	0.04	–0.16	–0.07	–0.03	0.08	0.16	0.03	–0.21	0.19
IPO primitive defenses	0.14	–0.02	0.15	0.19	0.33	0.35	0.27	–0.33	–0.30
IPO identity diffusion	0.02	–0.01	0.02	–0.04	0.24	0.36	0.23	–0.30	–0.14
IPO reality testing	0.08	0.08	0.05	0.20	0.26	0.17	0.30	–0.24	–0.42
**Variable**	**Cluster 2 (*n* = 9)**								
SCL GSI	–0.43	–0.31	–0.25	–0.12	–0.46	–0.34	*N*/*A*	0.20	0.10
STAI state anxiety	−0.85**	–0.50	–0.51	0.26	–0.09	0.04	*N*/*A*	–0.20	–0.43
STAI trait anxiety	–0.31	–0.48	–0.13	0.05	–0.11	0.17	*N*/*A*	–0.06	–0.19
STAXI state anger	0.10	0.44	0.18	–0.07	–0.23	–0.26	*N*/*A*	–0.01	0.33
STAXI trait anger	–0.22	–0.21	0.07	0.65	0.40	0.37	*N*/*A*	–0.33	–0.17
BDI	–0.63	0.05	–0.16	–0.03	–0.63	–0.51	*N*/*A*	–0.35	–0.17
IIP total	–0.45	–0.40	–0.12	–0.27	–0.46	–0.38	*N*/*A*	0.13	–0.03
IPO primitive defenses	0.07	–0.07	0.14	–0.37	–0.62	–0.33	*N*/*A*	0.03	0.14
IPO identity diffusion	0.01	–0.09	–0.19	–0.34	–0.66	–0.32	*N*/*A*	0.20	0.16
IPO reality testing	–0.46	–0.41	0.01	0.16	–0.33	–0.28	*N*/*A*	–0.28	–0.24

SCL, symptom checklist-90-revised; STAI, Spielberger state and trait anxiety inventory; STAXI, Spielberger state and trait anger inventory; BDI, beck depression inventory; IIP, inventory of interpersonal problems; IPO, inventory of personality organization; N/A, not applicable. *p < 0.05, **p < 0.01 (2-tailed).

#### Impact of Facial Affective Clusters on Treatment Outcome

**FIGURE 1:**
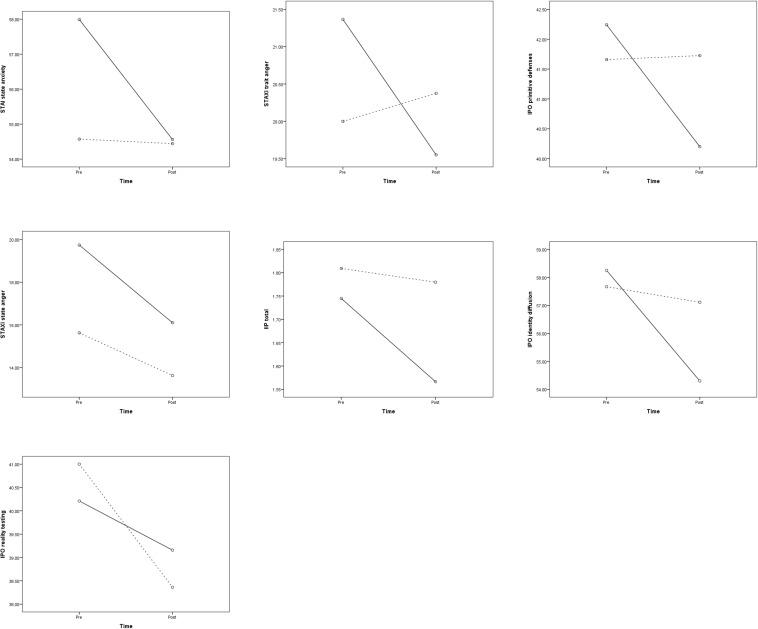
Interaction effects between time and cluster (solid line: Cluster 1; dotted line: Cluster 2) for Spielberger state and trait anxiety inventory (STAI) state anxiety, Spielberger state and trait anger inventory (STAXI) state and trait anger, inventory of interpersonal problems (IIP) total, and the three scales of the inventory of personality organization (IPO).

Table 5 presents the means and standard deviations of the study variables for the two clusters at pre and post, and Table 6 presents the results of the repeated measure ANOVAs. The results showed that there were two significant time effects, namely for SLC GSI and BDI. Furthermore, there were no significant time-by-cluster interaction effects and no significant effects between the clusters. However, due to the small sample size (as mentioned in the section “Materials and Methods”), we do focus on the effect sizes instead of the *p*-values. Looking at the effect sizes, the results of the ANOVAs showed that there were small to large time effects on all outcome variables, indicating that the psychopathology, interpersonal problems, and problematic personality organization reduced from pre to post. A large effect was found for the SCL GSI and medium effects for STAXI state anger, IIP total, IPO identity diffusion, and IPO reality testing. In 7 out of 10 variables, there were time-by-cluster interaction effects (see also Figure 1). A medium effect was found for the interaction with the STAXI trait anger, meaning that Cluster 1 showed reduced trait anger over time compared to Cluster 2. Small effects were found for the interactions with the STAI state anxiety, STAXI state anger, IIP total, and the three scales of the IPO indicating that Cluster 1 showed more reductions on these variables than Cluster 2, except for the scale IPO reality testing. For the IPO reality testing, Cluster 2 showed a bigger reduction compared to Cluster 1. In 5 out of 10 variables, there were also small effects between the clusters, namely for SCL GSI, STAI state anxiety, STAXI state anger, BDI, and IIP total. Specifically, Cluster 1 showed higher scores on the SCL GSI, STAI state anxiety, and STAXI state anger compared to Cluster 2. However, Cluster 2 showed higher scores on the BDI and IIP total compared to Cluster 1.

**TABLE 5:** Means and standard deviations of the study variables for the two clusters at pre and post.

Variable		Cluster 1 (*n* = 20)				Cluster 2 (*n* = 9)			
		Pre		Post		Pre		Post	
	Possible range	*M*	SD	*M*	SD	*M*	SD	*M*	SD
SCL GSI	0–4	1.48	0.58	1.33	0.74	1.34	0.77	1.12	0.62
STAI state anxiety	20–80	57.99	10.32	54.56	12.44	54.57	10.56	54.44	9.77
STAI trait anxiety	20–80	58.65	8.75	57.29	10.35	59.11	7.47	57.07	7.33
STAXI state anger	0–66	19.74	9.24	16.10	7.53	15.63	5.93	13.63	4.27
STAXI trait anger	0–66	21.37	6.99	19.55	6.45	20.00	6.21	20.38	5.60
BDI	0–63	26.52	9.86	21.54	13.68	29.09	7.12	23.65	12.28
IIP total	0–4	1.74	0.53	1.57	0.51	1.81	0.33	1.78	0.49
IPO primitive defenses	16–80	42.24	9.97	40.20	10.14	41.66	6.49	41.73	7.33
IPO identity diffusion	21–105	58.25	14.22	54.30	15.72	57.67	8.32	57.11	9.44
IPO reality testing	20–100	40.21	12.80	39.16	15.35	41.00	13.57	38.36	7.78

*SCL, symptom checklist-90-revised; STAI, Spielberger state and trait anxiety inventory; STAXI, Spielberger state and trait anger inventory; BDI, beck depression inventory; IIP, inventory of interpersonal problems; IPO, inventory of personality organization. n for Cluster 1 was 19 or 20. n for Cluster 2 was 8 or 9.*

**TABLE 6:** Results of the repeated measures ANOVAs for the study variables.

Source/Variable	SCL GSI				STAI state anxiety				STAI trait anxiety				STAXI state anger				STAXI trait anger			
	MS	*F*	*p*	$\eta_{\text{p}}^{2}$	MS	*F*	*p*	$\eta_{\text{p}}^{2}$	MS	*F*	*p*	$\eta_{\text{p}}^{2}$	MS	*F*	*p*	$\eta_{\text{p}}^{2}$	MS	*F*	*p*	$\eta_{\text{p}}^{2}$
Within																				
Time	0.42	4.22	0.049	0.14	39.28	0.45	0.51	0.02	35.91	1.40	0.25	0.05	91.03	3.14	0.09	0.11	5.94	0.96	0.34	0.04
Time*Cluster	0.01	0.10	0.75	0.00	33.80	0.39	0.54	0.01	1.44	0.06	0.81	0.00	7.73	0.27	0.61	0.01	13.72	2.22	0.15	0.08
Error (Time)	0.10				86.35				25.57				28.99				6.19			
Between																				
Cluster	0.38	0.46	0.50	0.02	38.73	0.24	0.63	0.01	0.18	0.00	0.97	0.00	124.25	1.39	0.25	0.05	0.84	0.01	0.92	0.00
Error	0.81				158.91				136.14				89.30				78.75			

**Source/Variable**	**BDI**				**IIP Total**				**IPO Primitive Defenses**				**IPO Identity Diffusion**				**IPO Reality Testing**			
	**MS**	***F***	***p***	**$\eta_{\text{p}}^{2}$**	**MS**	***F***	***p***	**$\eta_{\text{p}}^{2}$**	**MS**	***F***	***p***	**$\eta_{\text{p}}^{2}$**	**MS**	***F***	***p***	**$\eta_{\text{p}}^{2}$**	**MS**	***F***	***p***	**$\eta_{\text{p}}^{2}$**

Within																				
Time	309.98	6.61	0.02	0.20	0.12	2.28	0.14	0.08	12.13	0.69	0.41	0.02	63.00	2.53	0.12	0.09	41.58	2.18	0.15	0.08
Time*Cluster	0.60	0.01	0.91	0.00	0.06	1.17	0.29	0.04	13.82	0.79	0.38	0.03	35.76	1.44	0.24	0.05	7.67	0.40	0.53	0.02
Error (Time)	46.88				0.05				17.60				24.87				19.06			
Between																				
Cluster	62.42	0.29	0.59	0.01	0.22	0.49	0.49	0.02	2.75	0.02	0.89	0.00	15.40	0.05	0.83	0.00	0.00	0.00	1.00	0.00
Error	215.00				0.44				153.20				338.27				332.78			

*SCL, symptom checklist-90-revised; STAI, spielberger state and trait anxiety inventory; STAXI, spielberger state and trait anger inventory; BDI, beck depression inventory; IIP, inventory of interpersonal problems; IPO, inventory of personality organization; df, degree of freedom; MS, mean square; $\eta_{\text{p}}^{2}$, partial eta^2^, df for error (time) ranged between 25 and 27.*

## Discussion

The present study aimed to examine (a) the facial affective behavior in patients with a BPD, (b) whether these patients could be divided into clusters according to facial affective behavior, and (c) whether these clusters would influence the inpatient treatment outcome. In a first step, we examined general facial affective behavior. Corresponding to the results of Benecke and Dammann (2004), Renneberg et al. (2005), Buchheim et al. (2007), and Hepp et al. (2019), we found a high amount of negative facial affects such as disgust and contempt in our patients. The highest score was found for disgust, which seems to be the predominant emotion for these patients (Krause, 1998; Benecke and Dammann, 2004; Buchheim et al., 2007). Peham et al. (2015), who found high frequency of disgust expressions in different clinical groups and in healthy controls, argued that the facial configuration of disgust more generally expresses aversion to situations, other people, or self-aspects. Aversion is less specific than disgust, but nevertheless implies the wish to maintain or enlarge the distance between oneself and a negative stimulus. Presumably, patients with BPD are chronically in a mental state of being too close to something negative or threatening. The predominance of facial disgust expressions might contribute to the described interpersonal problems of these groups of patients by eliciting negative evaluation (Hepp et al., 2019). In a similar vein, Benecke et al. (2011) described a maladaptive cycle of easily triggered internal affective states (e.g., passive-negative affect, such as fear or despair) in patients with severe mental disorders based on problematic former relationship experiences. Specifically, patients try to “cope” with this inner situation by generating aggressive emotions (e.g., disgust) toward others. Aggressive emotions, in turn, prevent them from experiencing these passive-negative affective states and keep interaction partners, who indeed tend to withdraw from the patients, at a distance. The withdrawal of others leads to the experience of social rejection, which, in turn, intensifies these “problematic” wishes and thereby produces negative affect such as disgust once again (Ille et al., 2014). Intensified negative feelings are also closely related to maladaptive defenses and could therefore lead patients to deny their true inner states. Such defenses help to keep others at a distance and to maintain an illusory picture of healthiness because, from the patients’ point of view, only the behavior of others is problematic. On the other hand, the patients in this study showed a lot of social smile, which could be interpreted as a defense mechanism trying to keep a minimal interpersonal contact, despite of being forced to enlarge distance between self and objects (Buchheim et al., 2007). Fear and surprise were shown very rarely. The two emotions are partly very close in their facial expression. Both signal the need for information, whereby, in the case of fear, the evaluation of an object or situation as threatening has already been made, in the case of surprise, it is still open. According to the clinical theories, in which anxiety plays a prominent role, a higher rate of facial expressions of fear could have been expected. It could be that an automated regulation takes place here, in such a way that (unconscious) fear signals immediately activate a strong aversion, which is then expressed in the form of disgusting facial expressions (see Peham et al., 2015). This would explain why in both phobic and BPD patients, facial expressions of disgust rather than fear are the dominant facial expression of emotion. While in phobic patients facial disgust occurs specifically in response to the phobic object, in BPD patients, facial disgust or aversion is also predominant in social situations, so that interpersonal distortions quickly occur in these patients, since the patients actually need help internally, but the disgust expressions push the social partners away.

Our next finding refers to the results of the pilot study of Benecke and Dammann (2004). We were also able to divide our patients into two clusters: the Cluster 1 group with generally more facial affective behavior and significantly more anger, contempt, and disgust than patients in Cluster 2. Unlike in the study of Benecke and Dammann (2004), there were no significant differences regarding surprise and fear. Facial expressions of fear, surprise, and sadness in the patients of this study generally had low values, which makes it more unlikely to reach significance within group comparison.

The correlations between the facial affective behavior and the study variables showed that the general facial activity was positively correlated with state anger in Cluster 1, but negatively correlated with state anxiety in Cluster 2.

With regard to the treatment outcomes, the analysis of effects sizes (the *p*-values were statistically not significant) indicated that the psychopathology, interpersonal problems, and personality organization improved after 12 weeks of inpatient treatment, which is in line with previous studies (Sollberger et al., 2015; Dammann et al., 2016). Moreover, the results of the effect sizes seem to reveal that the persons of Cluster 1 benefitted more from the treatment in terms of reduced state anxiety, state and trait anger, interpersonal problems, and two aspects of the personality organization, namely primitive defenses and identity diffusion, compared to persons of Cluster 2. However, the persons of Cluster 2 seem to benefit more from the treatment in one aspect of personality organization, namely reality testing. In sum, it could be argued that BPD patients who showed more facial affective behavior seem to respond better to the inpatient treatment than BPD patients who showed less facial affective behavior, and that the latter could represent a more disturbed subgroup in terms of personality organization. However, it could also be argued that more disturbed patients or patients with less facial affective behavior have more room for change and therefore show greater treatment success.

In future studies, it might be interesting to examine the specific functions for each of the primary affects and how they impact the treatment outcome of BPD patients. For example, Bock (2011) and Bock et al. (2016) found different functions of aggressive facial behavior in BPD patients compared to patients with other disorders and to healthy controls. Finally, the results indicated that the two clusters seem to differ on the study variables that were rather state and not trait, strengthening the assumption that facial behavior should receive more attention in the treatment context. For instance, patients could be trained to consciously show more positive facial display in order to break the negative cycle between negative affect display and social withdrawal. This is in line with the broaden-and-build theory of positive emotions (Fredrickson, 1998, 2001) that posits that positive emotions broaden (rather than narrow) an individual’s thought-action repertoire and thus initiate upward spirals toward enhanced well-being. Positive emotions can also trigger this upward spiral of emotional well-being regardless of negative emotions (Fredrickson and Joiner, 2002). Positive psychotherapy has shown to be effective in reducing depressive symptoms (Seligman et al., 2006), and therefore, increasing positive emotions in BPD patients could be a promising way. The results on non-verbal synchrony between the patient and the therapist further support the importance of non-verbal behavior in the treatment context. For instance, Ramseyer and Tschacher (2011) found that higher non-verbal synchrony (i.e., body movement) between the patient and the therapist characterized psychotherapies with higher symptom reduction. In a similar vein, Ramseyer et al. (2020) examined non-verbal synchrony in BPD patients and healthy controls under oxytocin and placebo. The results showed that the controls displayed increased synchrony under the oxytocin condition, whereas the BPD patients did not.

There are several limitations to our exploratory study, without another clinical control group, reducing the generalizability of the results: the limited number of participants of the study and the lack of a non-borderline control group. In addition, in our sample, many BPD patients were diagnosed with a comorbid Axis I Disorder, which should be accounted for in future studies. Due to the small sample sizes, we interpreted the effect sizes instead of the *p*-values. These preliminary findings must therefore be interpreted with caution. We assessed facial expressivity in the context of a clinical interview (Kernberg-based “structural interview of personality organization”) as the stimuli used. Perhaps this specific interview elicits particular emotions? Negative emotional expressivity could also be interpreted in the context of an interview about clinically relevant difficulties within an inpatient sample. Of course, further research is needed to confirm our findings and to clarify open questions, e.g., the importance of facial expression to BPD functioning, and the relationship between change in symptoms with change in facial affect. Did treatment responders show any changes in facial affect? Another limitation of the current study is that we did not have detailed information about what kind of medications the patients received. It could be that certain medications influence the facial affective behavior more than others (e.g., less facial expression in patients who receive antipsychotics), which could account for the difference between the two clusters. This should be investigated in future studies. Many studies have given evidence that problems of BPD patients in social interactions seem to be linked to bias in facial emotion recognition or behavioral facial expression (Matzke et al., 2014; Brüne, 2016; Hauschild et al., 2018). However, this was a study of facial behavior, not recognition. It is not really clear how facial expression informs research on facial recognition. To conclude, our results of the effect sizes (although not significant) indicated that BPD patients with higher scores on the affective facial expressions may benefit more from the treatment. However, more research of facial affective behavior in BPD is needed.

## Data Availability Statement

The raw data supporting the conclusions of this article will be made available by the authors, without undue reservation.

## Ethics Statement

The studies involving human participants were reviewed and approved by Ethics Committee of Basel (EKBB). The patients/participants provided their written informed consent to participate in this study.

## Author Contributions

GD wrote the original draft and was the PI of the study. MR analyzed the data, wrote the result section, and worked on the original draft and the editing of the manuscript. CB analyzed with his team the facial expression data. AR collected the data together with DG-H. MW and JK were Co-PI of the study. MP helped in formulating the hypotheses of the study. JC helped with the statistical method. DG-H collected the data and co-analyzed them. All authors contributed to the article and approved the submitted version.

## Conflict of Interest

The authors declare that the research was conducted in the absence of any commercial or financial relationships that could be construed as a potential conflict of interest.
